# Short term monotherapy with GLP-1 receptor agonist liraglutide or PDE 4 inhibitor roflumilast is superior to metformin in weight loss in obese PCOS women: a pilot randomized study

**DOI:** 10.1186/s13048-015-0161-3

**Published:** 2015-06-02

**Authors:** Mojca Jensterle, Vesna Salamun, Tomaz Kocjan, Eda Vrtacnik Bokal, Andrej Janez

**Affiliations:** Department of Endocrinology, Diabetes and Metabolic Diseases, University Medical Centre Ljubljana, Zaloska 7, SI-1000 Ljubljana, Slovenia; Department of Obstetrics and Gynecology, Reproductive Unit, University Medical Centre Ljubljana, Zaloska 7, 1525 Ljubljana, Slovenia

**Keywords:** Liraglutide, Metformin, Obesity, PCOS, Roflumilast

## Abstract

**Objective:**

To evaluate whether liraglutide or roflumilast significantly affects body weight when compared to metformin in obese women with PCOS.

**Design/main outcome measure:**

A 12-week prospective randomized open-label study was conducted with 45 obese women with PCOS diagnosed by the ASRM-ESHRE Rotterdam criteria. They were randomized to metformin (MET) 1000 mg BID or liraglutide (LIRA) 1.2 mg QD s.c. or roflumilast (ROF) 500 mcg QD. The primary outcome was change in measures of obesity.

**Results:**

Forty-one patients (aged 30.7 ± 7.9 years, BMI 38.6 ± 6.0 kg/m^2^, mean ± SD) completed the study. Subjects treated with LIRA lost on average 3.1 ± 3.5 kg (*p* = 0.006), on ROF 2.1 ± 2.0 kg (*p* = 0.002) vs. 0.2 ± 1.83 kg in MET group. BMI decreased for 1.1 ± 1.26 kg/m^2^ in LIRA (*p* = 0.006), for 0.8 ± 0.99 kg/m^2^ in ROF (*p* = 0.001) vs. 0.1 ± 0.67 kg/m^2^ in MET. LIRA was superior to MET in reducing weight (*p* = 0.022), BMI (*p* = 0.020), waist circumference (*p* = 0.007). LIRA also resulted in decrease in VAT area (*p* = 0.015) and more favorable dynamics in glucose homeostasis during OGTT. ROF resulted in reduction of waist circumference (*p* = 0.023). In addition, ROF led to testosterone reduction (*p* = 0.05) and increase in menstrual frequencies (*p* = 0.009) when compared to baseline.

**Conclusion:**

Short-term monotherapy with liraglutide or roflumilast was associated with significant weight loss in obese PCOS. Liraglutide was superior to metformin, whereas roflumilast resulted in greater, yet not statistically significant, mean weight loss when compared to metformin. Reduction of body weight with liraglutide resulted in improvement of body composition.

**Trial registration:**

ClinicalTrials.gov NCT02187250.

## Background

Obesity is frequently present in women with polycystic ovary syndrome (PCOS) and aggravates the adverse features of the syndrome [1]. In overweight and obese patients with PCOS, lifestyle changes represent first-line treatment [2]. However, weight loss is frequently unsatisfactory with lifestyle changes alone or in combination with metformin and many women eventually regain weight [3]. In these women, the combination of lifestyle changes with identification of new effective and safe treatment options for weight reduction could be considered.

Pharmacological interventions on incretin system are a novel treatment of type 2 diabetes and proved valuable for weight loss in other obese populations [4]. In addition, obese women with PCOS who have not responded to lifestyle modification and metformin benefit from 12 week treatment with long-acting glucagon-like peptide (GLP)—1 receptor agonist liraglutide as an add-on therapy to metformin [5]. Experiences with the use of GLP-1 receptor agonist regarding weight loss as a primary outcome in PCOS are still very limited although an essential role of GLP-1 as a multi-targeting regulator of food intake has been recognized also in this population [6].

Less recognized distinct regulatory mechanisms related to the enhancement of GLP-1 mediated action through the inhibition of phosphodiesterase enzymes (PDE) 4 has recently became a reasonable focus of a potential new anti-obesity management. Roflumilast, the first drug specifically targeting PDE4, is well recognized as efficient treatment of chronic inflammatory diseases, primarily chronic obstructive pulmonary disease (COPD) [7, 8]. In addition, the use of roflumilast has shown positive metabolic effects on glucose homeostasis and weight reduction in newly diagnosed type 2 diabetes mellitus (T2DM) [9]. Furthermore, combined therapy of roflumilast and metformin significantly reduced body weight in obese PCOS when compared to metformin, primarily due to a loss of fat mass [10]. The observed beneficial metabolic outcomes of selective PDE4 inhibition by roflumilast are based on the interplay between the PDE4 and the regulation of GLP-1 [11].

No previous studies to date compared the effect of long acting GLP-1 receptor agonist liraglutide and selective PDE4 inhibitor roflumilast that is involved in GLP-1 release on body weight either in PCOS or any other obese population. The aim of this pilot study was to evaluate whether the monotherapy with liraglutide or roflumilast significantly affects body weight when compared to metformin in obese women with PCOS.

The study is registered on www.ClinicalTrials.gov as NCT02187250.

## Methods

### Study design

The study consists of a 12-week prospective randomized open-label design conducted with 45 obese women with PCOS diagnosed by ASRM-ESHRE Rotterdam criteria. All subjects had type A phenotype of PCOS including concomitant presence of a) hyperandrogenemia on either the biochemical or the clinical level, b) menses abnormalities and c) PCO morphology. They were eligible for enrollment if they were aged 18 years to menopause and obese (body mass index: BMI ≥ 30). Exclusion criteria was history of carcinoma or neuropsychiatric events, personal or familiar history of multiple endocrine neoplasia 2, significant cardiovascular, kidney or hepatic disease and the use of medications known to affect reproductive or metabolic functions within 180 days prior to study entry. Some of them were using oral contraceptives advised by their gynecologists more than 6 month before being recruited.

They were randomized to one of the three treatment arms: metformin (MET) 1000 mg BID or liraglutide (LIRA) 1.2 mg QD s.c. or roflumilast (ROF) 500 mcg QD. Liraglutide was initiated at a dose of 0.6 mg injected s.c. once per day and increased to 1.2 mg/day after 1 week. Metformin was initiated at a dose of 500 mg once per day and increased by 500 mg every 3 days up to 1000 mg BID. Patients on liraglutide were provided with glucose-monitoring devices and supplies and educated on their use. They were instructed to measure blood glucose levels at any signs and symptoms suggesting low blood glucose. Hypoglycemia was defined according to American Diabetes Association criteria as symptoms suggestive of low blood glucose confirmed by self-monitored blood glucose measurement below 3.9 mmol/l [12]. All women were instructed to report any side effects during the treatment. They were given a general advice on lifestyle intervention. A diet of 500–800 kcal/day reduction made up of 50 % carbohydrates, 20 % proteins and 30 % of fat with increased consumption of fiber, whole grains, cereals, fruits and vegetables along with at least 30 min of moderate intensity physical activity daily had been recommended to all women at the beginning of the study. During the study lifestyle intervention was not again actively promoted.

The primary outcome of the study was mean change in measures of obesity. Secondary outcomes included hormonal and metabolic changes. Post randomization and at study endpoint all patients underwent standard anthropometric measurements: height, weight, waist circumference, blood pressure, measurement of whole-body composition by a Hologic Dual Energy X-ray Absorptiometer (DXA), including visceral adipose tissue (VAT) area. BMI was calculated as the weight in kilograms divided by square of height in meters. Before randomization transvaginal ultrasound scans of the ovaries was performed by an experienced sonographer. The presence of PCO morphology was diagnosed by the presence of 12 or more follicles in each ovary measuring 2–9 mm in diameter and/or increased ovarian volume (>10 cm^3^).

A fasting blood was drawn for determination of glucose, insulin, luteinizing hormone (LH), follicle stimulating hormone (FSH), androstenedione, dehydroepiandrosterone sulphate (DHEAS), total and free testosterone (T) followed by a standard 75 g oral glucose tolerance test (OGTT) to assess glucose homeostasis. Glucose levels were determined using a standard glucose oxidase method (Beckman Coulter Glucose Analyzer, Beckman Coulter Inc CA, USA). Insulin was determined by immunoradiometric assay (Biosource Europe S.A., Nivelles, Belgium). LH and FSH were determined using an immunometric assay (Diagnostic Products Corporation, LA). Androstenedione and DHEAS were measured by specific double antibody RIA using 125 I-labeled hormones (Diagnostic Systems Laboratories, Webster, Tx). Total and free testosterone levels were measured by coated tube RIA (DiaSorin, S. p. A, Salluggia, Italy and Diagnostic Products Corporation, LA, respectively). Sex hormone binding globulin (SHBG) was determined with a chemiluminescent immunoassay (Immulite 2000 Analyzer, Siemens Healthcare, Erlangen, Germany). Intraassay variations ranged from 1.6 to 6.3 %, and interassay variations ranged from 5.8 to 9.6 % for the applied methods. Pre- and posttreatment samples from each patient were assayed in the same assay run.

Homeostasis model assessment (HOMA-IR) calculation was applied as a measure for insulin resistance (IR). Safety clinical assessment was performed at the beginning and week 4, 8 and 12 of the treatment period. Pregnancy was excluded by measuring β-human chorionic gonadotropin. Its measurement was repeated during the study whenever pregnancy was clinically possible. Women were advised to strictly use barrier contraception.

### Study approval

The study was approved by a National Medical Ethics Committee and conducted in accordance with the Declaration of Helsinki and Good Clinical Practice guidelines. Informed consent was obtained from all patients before participation.

### Statistical analysis

The primary endpoint of this study, for which a power calculation was used to determine the sample size, was mean change in weight. We based our calculations on a previous study with comparative treatment intervention [4]. It was determined that 12 patients would be needed per group in order to give a power of 80 % for the detection of a statistically significant difference (alpha = 0.05) of approximately 2.5 % in weight loss and estimated dropout rate of 15 %. The power estimate was performed with the online calculator at http://hedwig.mgh.harvard.edu/sample_size/size.html. A block randomization was used for subject assignment in random block sizes of 3, 6, and 9. A list of random numbers was excel generated and each random number corresponds with 1 of the 3 possible interventions (MET, LIRA, ROF).

Results are presented as mean ± standard deviation (SD). Normal data distribution was checked with the Shapiro-Wilk test. Differences at baseline between the treatment groups were checked with general linear model. The differences between treatment groups were checked and confirmed using ANOVA test. Treatment impact on the primary outcome measure was analyzed with evaluable patients data using repeated-measures linear mixed effects model with time points (baseline, 12 weeks) and therapeutic groups (MET arm, LIRA arm, ROF arm). Multiple testing of secondary outcomes was not performed. P value of less than 0.05 was considered statistically significant. Other than sample size calculation, all statistical analyses were performed using the SPSS 17.0 Statistical Software Package.

## Results

### Baseline results

The study enrolled 45 participants. Two MET treated patient discontinued the study, one because of diarrhea, one due to protocol violation, one LIRA treated because of nausea and one ROF treated because of headache. Forty-one patients (aged 30.7 ± 7.9 years, BMI 38.6 ± 6.0 kg/m^2^, mean ± SD) completed the study: 13 on MET, 14 on LIRA and 14 on ROF. There were no significant differences at baseline in any of the parameters between the treatment groups. Baseline characteristics of the study outcomes are provided in Tables 1 and 2.

**Table 1:** Baseline and 12-week post-treatment measures of obesity

	MET (*n* = 13)		LIRA (*n* = 14)		ROF (*n* = 14)		*P* values
	Baseline	After therapy	Baseline	After therapy	Baseline	After therapy	
Weight (kg)	108.3 ± 17.0	108.1 ± 17.5	102.8 ± 16.3	99.7 ± 17.2	111.1 ± 16.1	109.0 ± 16.4	T = 0.025 (L vs. M = 0.022); I (L = 0.006; R = 0.002)
BMI (kg/m^2^)	39.4 ± 6.9	39.3 ± 7.0	36.7 ± 5.6	35.6 ± 5.8	39.9 ± 5.4	39.1 ± 5.7	T = 0.023 (L vs. M = 0.020); I (L = 0.006; R = 0.001)
Waist circumference (cm)	120.5 ± 14.5	121.3 ± 13.2	115.7 ± 12.5	112.6 ± 12.9	123.0 ± 15.9	121.8 ± 16.1	T = 0.009 (L vs. M = 0.007); I (L = 0.009; R = 0.023)
VAT area (cm^2^)	131 ± 44.5	126.5 ± 48.3	160.3 ± 67.9	140.7 ± 60.8	157.3 ± 36.7	153.7 ± 21.9	I (L = 0.015)

For *p* values, T = overall effect after all treatments, R = ROF, L = LIRA, M = MET, I = interaction differences between treatment over trials

**Table 2:** Baseline and 12-week post-treatment metabolic and endocrine parameters

	MET (*n* = 13)		LIRA (*n* = 14)		ROF (*n* = 14)		*P* values
	Baseline	After therapy	Baseline	After therapy	Baseline	After therapy	
Glu 0 min OGTT (mmol/L)	4.8 ± 0.9	4.4 ± 1.6	5.1 ± 1.1	4.7 ± 0.7	5.1 ± 0.5	5.0 ± 0.4	I (L = 0.048)
Insulin 0 min OGTT (mU/L)	15.5 ± 6.0	12.7 ± 7.0	16 ± 9.3	14.5 ± 9.4	21.0 ± 7.7	21.0 ± 8.7	NS
HOMA-IR	3.3 ± 1.2	2.5 ± 1.9	3.8 ± 2.8	3.2 ± 2.4	4.5 ± 1.7	4.6 ± 2.1	NS
Total T (nmol/L)	1.7 ± 1.1	1.5 ± 1.2	1.7 ± 0.7	1.5 ± 0.6	1.8 ± 0.5	1.5 ± 0.6	I (R = 0.050)
Free T (nmol/L)	3.6 ± 2.6	4.3 ± 3.0	3.7 ± 2.0	3.2 ± 2.0	4.0 ± 2.2	4.0 ± 2.3	NS
SHBG (nmol/L)	28.6 ± 11.1	31.5 ± 5.6	30.5 ± 9.9	32.8 ± 14.2	27.0 ± 11.4	29.1 ± 12.1	NS
FAI	7.6 ± 8.0	4.8 ± 3.6	6.5 ± 3.7	5.4 ± 2.9	8.3 ± 5.9	6.1 ± 4.6	I (R = 0.016)
Androstenedione (nmol/L)	8.9 ± 3.8	10.7 ± 7.0	8.7 ± 3.4	9.0 ± 3.9	9.5 ± 2.5	9.4 ± 3.9	NS
DHEA-S (μmol/L)	5.9 ± 2.5	6.8 ± 3.5	6.6 ± 3.3	6.1 ± 2.4	5.6 ± 2.7	5.2 ± 1.9	NS

For *p* values, T = overall effect after all treatments, R = ROF, L = LIRA, M = MET, I = interaction differences between treatment over trials

*NS* not significant

### Measures of obesity

Subjects treated with LIRA lost on average 3.1 ± 3.5 kg (*p* = 0.006), on ROF 2.1 ± 2.0 kg (*p* = 0.002) vs 0.2 ± 1.83 kg weight loss in MET group (*p* = 0.735). BMI decreased for 1.1 ± 1.26 kg/m^2^ in LIRA (*p* = 0.006), for 0.8 ± 0.99 kg/m^2^ in ROF (*p* = 0.001) vs 0.1 ± 0.67 kg/m^2^ in MET (*p* = 0.731). LIRA was superior to MET in reducing weight (*p* = 0.022), BMI (*p* = 0.020) and waist circumference (*p* = 0.007). Roflumilast resulted in greater, yet not statistically significant, mean weight loss when compared to metformin (*p* = 0.203). Although the mean weight loss was greater in the LIRA than in the ROF arm the difference was not statistically significant (*p* = 0.992). LIRA resulted in significant decrease in VAT area (*p* = 0.015). Both LIRA and ROF were associated with waist circumference reduction when compared to baseline (*p* = 0.009 and *p* = 0.023, respectively). The mean pre-and post-treatment measures of obesity are presented in Table 1.

### Metabolic parameters

HOMA-IR decreased in all treatment arms, although the between treatment difference was not statistically significant yet. There was a statistically significant within-treatment reduction from baseline to last visit in fasting glucose levels and glucose at 30 (from 8.2 ± 2.4 to 7.7 ± 2.1 mmol/l, *p* = 0.028) and 120 min (from 6.7 ± 2.9 to 5.4 ± 1.9 mmol/l, *p* = 0.050) of OGTT in LIRA treated women. Liraglutide was superior to metformin in reducing glucose at 120 min of OGTT (*p* = 0.041). The mean pre-and post-treatment values of fasting glucose, fasting insulin and HOMA-IR are presented in Table 2.

### Endocrine parameters

At 12 weeks a significant total T and Free Androgen Index (FAI) reduction were noted in ROF arm when compared to baseline. No statistically significant differences were found in free T, SHBG, androstenedione, DHEAS (Table 2), or in LH and FSH, neither over time nor when analyzing it separately by therapeutic arm.

### Changes in menstrual pattern

Menstrual frequency increased with all treatments. The increase was shown as being slightly greater in patients treated with ROF (from 0.57 ± 0.40 to 0.88 ± 0.20 per month, *p* = 0.009), compared with MET (from 0.74 ± 0.30 to 0.92 ± 0.20 per month, *p* = 0.090) and LIRA (from 0.62 ± 0.30 to 0.74 ± 0.30, *p* = 0.165). However, the between-treatment differences were not statistically significant yet.

### Adverse events

The most commonly reported adverse events in MET group were diarrhea (4/14) and nausea (4/14) that resolved in the first week for 3/14 subjects and within 4 weeks of the study onset for the 1/14 woman. Adverse events associated with LIRA were nausea (4/14), obstipation (2/14), diarrhea (1/14), headache (1/14) and insomnia (1/14). In the ROF group, 5/14 subjects had mild gastrointestinal problems (nausea and diarrhea), 2/14 had mild headache and 1/14 reported mild depression in the last month of the study. Nausea in LIRA arm was present up to 3 days when liraglutide was initiated at a dose of 0.6 mg injected s.c. once per day and if present reappeared for 2 to 3 days when the dose was increased to 1.2 mg/day after 1 week. It was not accompanied with vomiting. Nausea in ROF arm was more persistent when compared to MET yet it was mild and not accompanied with vomiting. Some subjects in all treatment groups had multiple side effects. No side effect was reported by 10/14 women in MET arm, 8/14 in LIRA arm and 8/14 in ROF arm. Hypoglycemic event was not reported in any group. The injection regimen of LIRA did not impair adherence or cause significant withdrawal over ROF and MET during treatment.

## Discussion

The comparison of long-acting GLP-1 receptor agonist liraglutide or selective PDE4 inhibitor roflumilast versus metformin on changes of measures of obesity have not yet been evaluated in women with PCOS or any other obese population. To our knowledge, this is the first study to date demonstrating that in a short period of time liraglutide was significantly more effective than metformin regarding weight loss and improvement of body composition in obese PCOS women. Roflumilast resulted in greater, yet not statistically significant, mean weight loss when compared to metformin. In addition, liraglutide treatment was followed with within-treatment favorable improvements in glucose homeostasis during OGTT and in significantly greater reduction of glucose at 120 min of OGTT when compared to metformin, while roflumilast resulted in testosterone reduction and increase in menstrual frequencies when compared to baseline.

Liraglutide resulted in the between-treatment difference of about 3 kg weight lost versus metformin. Although metformin is still widely used in the management of PCOS due to its observed curative and potentially preventive clinical benefits, including body weight reduction and improvement of fat distribution, recent guidelines developed by the Endocrine Society recommended its use only in women with PCOS who are already undergoing lifestyle treatment and do not have improvement in impaired glucose tolerance and in those who have normal weight, but still have impaired glucose tolerance [13]. In fact, its effect on weight reduction and fat distribution in PCOS remains controversial. In accordance with our study, most studies and meta-analysis concluded that as a mono-therapy, metformin does not significantly reduce body weight, although some studies have reported some marginal benefit in longer observation period [14, 15].

Until today, only few studies have addressed the possible use of GLP-1 receptor agonists in women with PCOS, but all with some important methodological limitations regarding the evaluation of their effect on body weight. We previously conducted a study with obese PCOS women who had been pretreated with metformin and had lost less than 5 % of body weight in 6 months with metformin before recruitment. In that study, one group was kept on metformin for 12 more weeks while comparing it for weight loss with other two groups taking liraglutide alone or liraglutide in combination to metformin. Patients on combined treatment lost on average 6.5 kg compared with a 3.8 kg loss in LIRA group, with the between-treatment difference of about 5 and 3 kg, respectively, when compared to metformin mono-therapy [5]. The results are perfectly in line with the findings of this study. However, the main issue of our previous study was inclusion of non-responders for the outcome of interest that could have brought bias from the beginning. Apprised of that limitation the present study was conducted with a much better and fair design that included drug naïve patients. Furthermore, an average weight reduction of 3.0 kg, yet achieved in a longer period of observation and with larger dosage of 1.8 mg QD s.c., was observed also in a 24 week-study that reported the potential impact of liraglutide on markers of liver fibrosis in PCOS [16]. However, the study was not designed to evaluate the change in measures of obesity as a primary outcome of interest. As opposed to long acting GLP-1 agonist liraglutide, the only report using short acting GLP-1 agonist exenatide in treatment-naive overweight patients with PCOS was a 24 week-study conducted to evaluate change in menstrual frequency whereas change in body weight was pre-specified as secondary outcome. Combined treatment with exenatide and metformin was superior to exenatide and metformin mono-therapies resulting in an average weight reduction of 6 kg in combined group compared to 3.2 kg in the exenatide, and of 1.6 kg in metformin group [17]. Weight reduction in our study is of comparable magnitude to the exenatide group, but achieved in a shorter period of time. In addition, we observed small drop out in our study as opposed to relatively large drop out in the study with exenatide, which was probably conditioned by the choice of GLP-1 receptor agonist. It has been demonstrated in individuals with type 2 diabetes mellitus that liraglutide caused less adverse events and therapy discontinuation than exenatide [18].

Contrary to liraglutide acting as a GLP-1 receptor agonist, selective PDE4 inhibitor roflumilast interacts to GLP- mediated effect through completely different pathways. It is involved in the PDE4 regulation of signaling cascades linked to GLP-1 release. In rodent model roflumilast enhanced plasma GLP-1 levels up to 2.5 –fold [11]. In clinical studies, roflumilast was associated with a weight decrease of about 2 kg versus placebo in 12 months in patients with chronic obstructive pulmonary disease (COPD) [7, 8] and with mean weight change of about 2 kg in 12 weeks versus placebo in patients with newly diagnosed type 2 diabetes mellitus without COPD [9]. We recently reported that in obese women with PCOS, roflumilast added to metformin was superior to metformin alone in reducing mean body weight after 12 weeks, with the between treatment difference of about 5 kg [10]. Referring to the patients’ history on pretreatment with metformin we were cautioned that the study did not have a proper control to draw definitive conclusions solely about the effects of roflumilast in PCOS. Considering the innovative concept that grasps selective PDE4 inhibition as a potential new therapeutic target in obesity associated populations, in the present study we used an upgraded design to further elucidate the potential effect of roflumilast in treatment-naïve obese PCOS population. Treatment with roflumilast resulted in the between-treatment difference of about 2 kg weight lost versus metformin, which is in accordance with the study of the same observational period in patients with newly diagnosed type 2 diabetes mellitus [9]. However, the weight reduction with both liraglutide and roflumilast in the present study was of lesser magnitude when compared to the studies in PCOS population using combined therapies, including combined treatment of GLP-1 receptor agonist [5, 17] and metformin or combination of roflumilast and metformin [10], which might suggest a possible additive role of metformin to GLP-1 mediated effect [19, 20].

Furthermore, the results of this study indicate that liraglutide was superior to roflumilast regarding improvements of measures of obesity and metabolic profile, not being demonstrated with roflumilast. Previously, effects of roflumilast on glucose metabolism has been studied in a 12 week, randomized, double blind, placebo controlled study of patients with newly diagnosed type 2 diabetes mellitus without COPD. Over 12 weeks, HbA1C levels declined substantially in the roflumilast group compared with placebo [9]. The beneficial impact of roflumilast on metabolic parameters has been explained by the same mechanisms mediated through its elevating effect on the GLP-1 incretin hormone levels as being hypothesized to be responsible for its effect on body weight reduction [9]. No study to date directly compared these two therapies and we might only reasonably speculate that the direct enhancement of GLP-1 axis with GLP-1 receptor agonists is more potent than GLP-mediated effect through PDE4 regulation. However, this was a pilot study, not powered for all possible relevant metabolic outcomes, but powered specifically for weight loss.

Surprisingly, despite the superiority of liraglutide in weight reduction and glucose homeostasis, roflumilast resulted in total testosterone and FAI reduction and increase in menstrual frequencies not being demonstrated in liraglutide arm. These observed beneficial trends that obviously went beyond weight reduction effect might be related to the expression of PDE4 enzymes in ovaries and the recognized involvement of PDEs in steroidogenesis. A potential role of roflumilast in direct regulation of ovarian steroidogenesis and in the pathophysiology of PCOS could not be excluded [21, 22]. The improvement of androgen profile tended to be slightly greater also in PCOS patients treated with roflumilast added to metformin in our previous study [10]. Clearly, due to generally known methodological problem for assessment of androgens in PCOS, relatively small study sample size and lack of any data on roflumilast specific effects on PCOS treatment outcomes, our observations in both studies so far do not allow more than a speculation in this regard.

The present study has several limitations. Number of patients in each treatment group was small, partially at the expense of the originality of the comparative design including three treatment arms. The 12-week observation period was too short to assess the efficacy and safety of liraglutide and roflumilast as weight loss drugs in obese women with PCOS. The short-term design was conducted mainly due to insufficient longer-term safety data of liraglutide and roflumilast in women that might still wish to conceive. The importance of lifestyle intervention was introduced at the beginning of the study but not actively promoted over the course of the study, yet such setting had the advantage of being similar to the daily practice. In addition, the open label nature of the study might be further potential limitation with this regard, as women in the LIRA arm would know they were given a drug that has been mentioned in the media as an anti-obesity drug. This could have resulted in a higher motivation to adhere to the lifestyle intervention. However, the main strength of this pilot study was the original concept that grasps inclusion of two agents acting through the GLP-1 axis that had not yet been compared either in PCOS or any other obese population.

## Conclusions

Short-term monotherapy with liraglutide or roflumilast was superior to metformin in weight loss in obese PCOS women. Reduction of body weight resulted in improvement of body composition. In addition, liraglutide was associated with beneficial effects on glucose homeostasis, whereas roflumilast resulted in total testosterone reduction and improvement of menstrual frequencies. Considering the recognized role of incretin system and potential role of PDE 4 signaling pathways in the pathophysiology of PCOS further explorations in larger studies of longer duration are needed to assess the effects of both agents in this population providing the possible basis for the clinical approach that could tailor treatment choices to the specific needs of the patient.

The role of metformin in the management of obesity remains unsatisfactory. Following recent clinical practice guidelines metformin should be used in women with PCOS who are already undergoing lifestyle treatment and do not have improvement in impaired glucose tolerance or in those who have normal body weight but still have impaired glucose tolerance [13]. However, the potential additive efficacy of novel anti-obesity treatment options targeting incretin system in combination to metformin should be a focus of further investigation in PCOS population.

## Abbreviations

PCOS: Polycystic ovary syndrome
GLP: Glucagon-like peptide
PDE: Phosphodiesterase enzyme
ASRM-ESHRE: American Society for Reproductive Medicine—European Society of Human Reproduction and Embryology
MET: Metformin treatment arm
LIRA: Liraglutide treatment arm
ROF: Roflumilast treatment arm
DXA: Dual Energy X-ray Absorptiometer
VAT: Visceral adipose tissue
LH: Luteinizing hormone
FSH: Follicle stimulating hormone
DHEAS: Dehydroepiandrosterone sulphate
T: Testosterone
OGTT: Oral glucose tolerance test
HOMA: Homeostasis model assessment
IR: Insulin resistance
COPD: Chronic obstructive pulmonary disease
